# Mining of vaccine-associated IFN-γ gene interaction networks using the Vaccine Ontology

**DOI:** 10.1186/2041-1480-2-S2-S8

**Published:** 2011-05-17

**Authors:** Arzucan Özgür, Zuoshuang Xiang, Dragomir R Radev, Yongqun He

**Affiliations:** 1 Faculty of Computer and Informatics, İstanbul Technical University, Maslak, İstanbul 34469, Turkey; 2 Department of Electrical Engineering and Computer Science, University of Michigan, Ann Arbor, MI 48109, USA; 3 Unit for Laboratory Animal Medicine, University of Michigan, Ann Arbor, MI 48109, USA; 4 Department of Microbiology and Immunology, University of Michigan, Ann Arbor, MI 48109, USA; 5 Center for Computational Medicine and Bioinformatics, University of Michigan, Ann Arbor, MI 48109, USA; 6 School of Information, University of Michigan, Ann Arbor, MI 48109, USA

## Abstract

**Background:**

Interferon-gamma (IFN-γ) is vital in vaccine-induced immune defense against bacterial and viral infections and tumor. Our recent study demonstrated the power of a literature-based discovery method in extraction and comparison of the IFN-γ and vaccine-mediated gene interaction networks. The Vaccine Ontology (VO) contains a hierarchy of vaccine names. It is hypothesized that the application of VO will enhance the prediction of IFN-γ and vaccine-mediated gene interaction network.

**Results:**

In this study, 186 specific vaccine names listed in the Vaccine Ontology (VO) and their semantic relations were used for possible improved retrieval of the IFN-γ and vaccine associated gene interactions. The application of VO allows discovery of 38 more genes and 60 more interactions. Comparison of different layers of IFN-γ networks and the example BCG vaccine-induced subnetwork led to generation of new hypotheses. By analyzing all discovered genes using centrality metrics, 32 genes were ranked high in the VO-based IFN-γ vaccine network using four centrality scores. Furthermore, 28 specific vaccines were found to be associated with these top 32 genes. These specific vaccine-gene associations were further used to generate a network of vaccine-vaccine associations. The BCG and LVS vaccines are found to be the most central vaccines in the vaccine-vaccine association network.

**Conclusion:**

Our results demonstrate that the combined usages of biomedical ontologies and centrality-based literature mining are able to significantly facilitate discovery of gene interaction networks and gene-concept associations.

**Availability:**

VO is available at: http://www.violinet.org/vaccineontology; and the SVM edit kernel for gene interaction extraction is available at: http://www.violinet.org/ifngvonet/int_ext_svm.zip

## Introduction

Interferon-gamma (IFN-γ) is one of the most important endogenous regulators of immune responses [1]. IFN-γ is vital in immune defense against infectious diseases, inflammatory conditions, tumor, and autoimmune diseases. For example, mice lacking either IFN-γ or its receptor have increased susceptibility to the infections of bacterial and viral pathogens [2]. It also regulates various immune responses that are often critical for induction of protective immunity generated by vaccines [1-3].

In our recent study, a novel literature-based discovery (LBD) approach based on Natural Language Processing (NLP) and network centrality analysis was applied to identify genes related to human IFN-γ (Gene symbol: IFNG) and potentially important for vaccine development [3]. Initially, a generic IFNG gene interaction network was built by automatically extracting the interactions of IFNG and other genes using abstracts from all articles in PubMed. A subnetwork was also generated by including the interactions extracted from vaccine-related sentences. Four network centrality metrics (i.e., degree, eigenvector, closeness, and betweenness) were further calculated to rank the genes in the constructed networks. By comparing the resulting generic IFNG network and the vaccine-specific subnetwork, many new observations and hypotheses were generated [3].

It is possible to further improve the literature-based network discovery by applying biomedical ontologies. A biomedical ontology represents the consensus-based controlled vocabularies of terms and relations which are logically formulated to promote intelligent information retrieval and modeling. The Vaccine Ontology (VO; http://www.violinet.org/vaccineontology) is a community-based ontology in the domain of vaccine and vaccination [4]. VO has classified a large number of existing vaccines in licensed use, on trial, or in research. Each subclass in VO has an “is_a” relationship with its parent class. This ensures that all vaccine subclasses (*e.g.*, the Bacillus Calmette-Guérin vaccine strain or BCG) can be included when a parent class (*e.g.*, “*Mycobacterium tuberculosis* vaccine” or “vaccine”) is searched in literature mining. In addition, VO includes many machine-readable annotations of various vaccines using the Web Ontology Language (OWL). For example, a vaccine’s quality (*e.g.*, live vs. inactivated) and components (*e.g.*, antigen and adjuvant) are defined in VO. These annotations can be processed by an ontology reasoner for automated reasoning. Currently, VO contains more than 500 vaccine names.

In this study, we incorporated the VO support to our LBD method. We hypothesized that the application of VO will increase our centrality-based literature retrieval of IFN-γ and vaccine-mediated gene interaction networks. Our results indicate VO significantly increases the retrieval of the IFNG-vaccine network and provides new insights and hypotheses for future investigations.

## Methods

The detailed literature-based network discovery methods were described in our recent publication [3]. Here we summarize the basis of the method with a focus on emphasizing the new application of VO in this approach.

### Literature corpus

All article abstracts and their titles available in PubMed (http://www.ncbi.nlm.nih.gov/pubmed/) were used. The sentences of the titles and abstracts were obtained from the BioNLP database in the National Center for Integrative Biomedical Informatics (NCIBI; http://ncibi.org/).

### Gene name identification and normalization

The machine learning-based software Genia Tagger (http://www-tsujii.is.s.u-tokyo.ac.jp/GENIA/tagger/) was used to identify the gene names in the sentences [5]. A dictionary-based approach was also used to normalize the gene names tagged by Genia Tagger so that each gene is represented by a single node in the interaction network. Only human genes were studied in this research. The HUGO Gene Nomenclature Committee (HGNC) database (http://www.genenames.org/) was used as the dictionary for human gene names and their synonyms. Each tagged human gene name was unified with its corresponding approved gene symbol.

### Literature mining

Sentences with at least two gene names and an interaction keyword(s) (*e.g.*, interacts, binds, activates, and etc.) were selected. In total more than 800 interaction keywords were manually collected and used in this study (the interaction keywords are available at: http://www.violinet.org/ifngvonet/interaction_keywords.txt). To extract the gene pairs that are stated as interacting in the sentences, we used a method based on machine learning and dependency parsing. Unlike a syntactic parse (which describes the syntactic constituent structure of a sentence), the dependency parse of a sentence captures the semantic predicate-argument relationships among its words. The nodes of a dependency parse tree represent the words of a sentence and the edges represent the types of the dependencies among the words such as subject, object and modifier. We obtained the dependency parse trees of the sentences using the Stanford Parser [6] and extracted the shortest dependency path between each pair of genes in a sentence. Our motivating assumption is that the shortest path between two gene names in a dependency tree is a good description of the semantic relation between them in the corresponding sentence.

**Figure 1:**
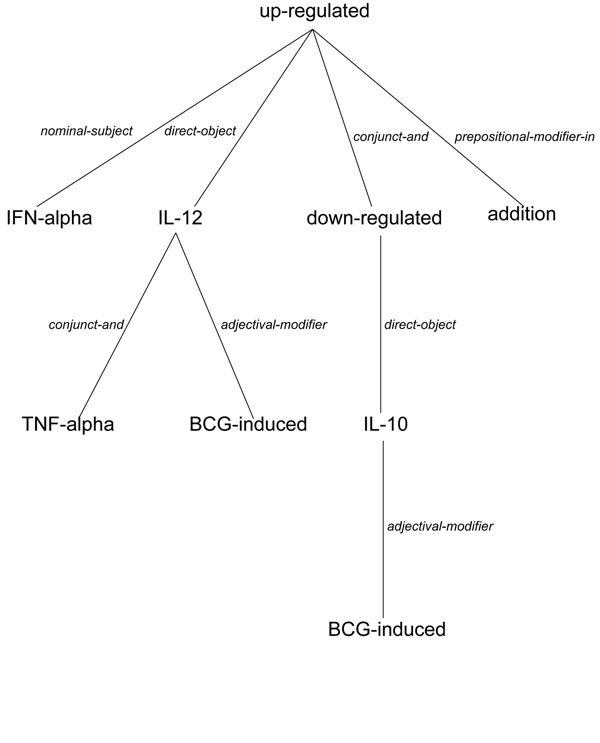
The dependency parse tree of the sentence “In addition, IFN-alpha up-regulated BCG-induced IL-12 and TNF-alpha and down-regulated BCG-induced IL-10”.

Figure 1 shows the dependency parse tree that we obtained for the sentence “In addition, IFN-alpha up-regulated BCG-induced IL-12 and TNF-alpha and down-regulated BCG-induced IL-10” from [7]. The interaction keywords in the sentence are “up-regulated”, “induced”, and “down-regulated”. This sentence contains four genes (IFN-alpha, IL-12, TNF-alpha, and IL-10), which means there are six different gene pairs. The sentence describes an interaction between the following three gene pairs in the context of the BCG vaccine (the dependency paths connecting the gene pairs are also provided).

• IFN-alpha & IL-12: *nominal-subject up-regulated direct-object*

• IFN-alpha & TNF-alpha: *nominal-subject up-regulated direct-object IL-12 conjunct-and*

• IFN-alpha & IL-10: *nominal-subject up-regulated conjunct-and down-regulated direct-object*

On the other hand, the sentence does not state direct interactions between the other three gene pairs: IL-12 & TNF-alpha, IL-12 & IL-10, and TNF-alpha & IL-10.

We defined an edit distance-based kernel function among these dependency paths and used support vector machines (SVM) to classify each path as describing an interaction between the gene pair or not. Edit distance between two dependency paths is defined as the number of term insertion, deletion, and substitution operations needed to transform the first path to the second. For example, to transform the path between IFN-alpha & IL-12 to the path between IFN-alpha & TNF-alpha, we need to perform two insertion operations (i.e., insert “IL-12” and insert “conjunct-and” to the first path).

The edit distance measure between two dependency paths p_i_ and p_j_ is converted to a similarity function as follows.

This dependency path similarity measure was integrated as a kernel function to SVM by plugging it in the SVM^light^ package (http://svmlight.joachims.org/) [8].

This interaction extraction approach was introduced in [9] and was shown that it achieves the state-of-the-art results (55.61% F-score performance for the AIMED data set (ftp://ftp.cs.utexas.edu/pub/mooney/bio-data/) and 84.96% F-score performance for the CB data set).

### Extraction of vaccine terms from VO

Specific vaccine terms were extracted from VO. Those vaccine terms that contain the name “vaccine” are not used in this study, since the term “vaccine” and its variants are explicitly included in the query for selecting the vaccine-related sentences. In total 186 vaccines were obtained from VO for this analysis (Additional file 1). These terms are the bottom-level terms of the ontology hierarchy under the term “vaccine”. The remaining vaccine terms in VO contain the word “vaccine” or its variants in their vaccine names. The inclusion of these terms does not help the identification of additional genes that interact with vaccines. Therefore, these terms were not used in this study. To maximize the searching capability, all synonyms of selected vaccine names shown in VO are included.

VO is used in two steps in this research. First, the list of 186 commercially used vaccines was directly extracted from VO, allowing our VO-based improvement on the extraction of IFNG and vaccine network. Secondly, the rich semantic constructs in the VO OWL format (*e.g.*, necessary and sufficient conditions) provide an effective approach for us to infer the subclasses of additional parent terms (*e.g.*, “inactivated vaccines”). These parent terms were then further explored in the content of IFNG and vaccine network.

### Centrality and ontology-based analysis of literature mined networks

Our centrality method calculates four different types of centralities: degree centrality (the number of neighbors of a node), eigenvector centrality (function of the centralities of a node’s neighbors), closeness centrality (inverse sum of the distances from the node to the other nodes in the network), betweenness centrality (the proportion of the shortest paths between all the pairs of nodes that pass through the node in interest) [10]. Different centralities measure different levels of importance. In degree centrality a node is considered important if it is connected to many other nodes in the network. In contrast to degree centrality, in eigenvector centrality each neighbor does not contribute equally to the centrality of a node. A node is considered more important if it is connected to many “central” nodes. In other words, besides the quantity of the connections of a node, their quality is also taken into account. In closeness centrality, a node is more important if its total distance to the other nodes in the network is smaller, whereas in betweenness centrality the importance of a node is higher if it occurs on many shortest paths between other nodes. Each of the four centrality measures is important to identify a specific role of a node in a specific network.

In this study the IFNG interaction network was analyzed from graph centrality perspective. IFNG and its neighbors are represented as nodes and there is an edge between two genes if an interaction between them has been extracted from the literature. The IFNG-vaccine subgraph of this network contains only the interactions that have been extracted from sentences that contain the term “vaccine” (or its variants like “vaccines”, “vaccination”, and “vaccinated”). There are many vaccine-related sentences in the literature where the term “vaccine” or its variants do not occur. Consider our example sentence (Figure 1) “In addition, IFN-alpha up-regulated BCG-induced IL-12 and TNF-alpha and down-regulated BCG-induced IL-10.” from [7]. The term “vaccine” or its variants occur neither in the sentence nor in the abstract. However, this sentence is vaccine-related, since “BCG” (Bacillus Calmette-Guerin) is a licensed tuberculosis vaccine. The “BCG” vaccine is included in the VO.

The VO specific IFNG network (IFNG-vaccine-VO), was created by extending the IFNG-vaccine network through including the interactions extracted from sentences that contain one of the specific vaccine names from the VO. Therefore, the edges in this subgraph are all vaccine specific. Comparative analysis of these IFNG and vaccine associated networks with or without the VO support helps us to understand the genes and interactions that play important roles in the IFNG and various vaccines. Since IFNG is one of the most important immune factors and critical for vaccine development, we hypothesized that genes central in the generic IFNG, IFNG-vaccine, and IFNG-vaccine-VO networks might be important for vaccine development.

To identify the associations of the most central genes with the specific vaccines in VO, a gene-vaccine co-occurrence analysis using all the abstracts in PubMed was performed. A gene is considered to be associated with a specific vaccine in VO if they have occurred in the same abstract. Furthermore, using the gene-vaccine association data, a vaccine-vaccine association network was generated. The nodes of this network are specific vaccines in VO. Two vaccines are connected with an edge if they share at least one central gene (i.e., co-occur with at least one central gene in an abstract). Once the vaccine-vaccine association network was generated, the same centrality method was used to compute the four centrality scores.

In many networks, nodes appear to form communities. While there are many edges between nodes within a community, there are fewer edges between communities. We applied the modularity-based community detection algorithm proposed in [11] to identify the communities among different vaccines in the vaccine-vaccine association network. Modularity is the difference between the fraction of all edges within communities and the expected value of the same quantity in a random graph of the same size. It is assumed that the higher the modularity value, the better the community divisions are. The algorithm in [11] tries to optimize the modularity of the network using a greedy approach. It starts with each node in a separate community. In each step, the two communities that produce the highest modularity are merged.

## Results

This study is an extension of previous IFNG and IFNG-vaccine network analyses [3]. The primary focus of this extended study is on the potential enhanced network retrieval based on the VO.

### Discovery of three different IFNG networks

**Figure 2:**
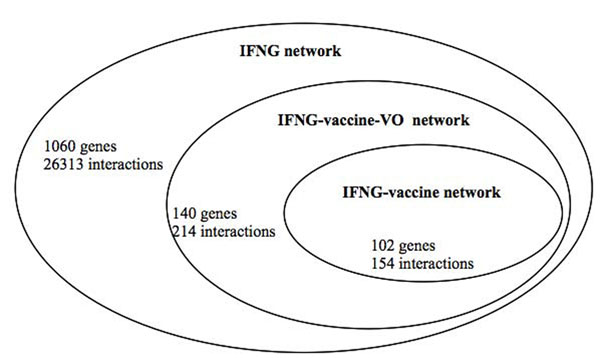
Three layers of IFNG-associated gene networks.

Three layers of IFNG networks were identified (Figure 2). The largest general IFNG network includes 1060 nodes (genes including IFNG and its neighbors) linked by 26313 edges (interactions). The smallest network is the IFNG-vaccine network as defined above. This network contains 102 genes and 154 interactions. The second largest IFNG-vaccine-VO network contains the small network and also genes and interactions associated with specific VO vaccine terms or their synonyms (*e.g.*, tuberculosis vaccine BCG). The intermediate contains 140 genes and 214 interactions. Therefore, the application of VO allows discovery of 38 more genes and 60 more interactions. These new genes and interactions were not identified if only the term vaccine (or its variants) were used.

### List of genes for vaccines or specific VO vaccine terms are predicted and sorted by centrality analyses

Figure 2 provides the general numbers of the different IFNG networks. To make more specific analysis, the most central genes (the genes ranked among the top 20 by at least one of the four centrality measures) are analyzed in more detail in Table 1. Since the networks are specific to IFNG, this gene is trivially ranked highest by all the centrality measures. Therefore, it is not included in the rankings. The remaining most central genes (a total of 32 genes) are predicted to be associated with IFNG and relevant for the general vaccine or specific vaccine term(s). Literature evidence was manually curated for the vaccine development relatedness (Reference column in Table 1) of these genes.

**Table 1:** Predicted 32 genes related to IFN-γ and vaccine networks.

Gene	Degree	Eigenvector	Betweenness	Closeness	Reference (PMID)
IL2	+	+	+	+	8459207
TNF	+	+	+	+	16446013
IL10	+	+	+	+	10930151
IL6	+	+	+	+	10225849
IL4	+	+	+	+	8519092
CSF2	+	+	+	+	19459853
IL8	+	+	+	+	11378044
IL5	+	+	-	+	11138639
NFKB1 *	+	+	+	+	16971487
IL13	+	+	+	+	12232042
CD4	+	+	+	+	17298856
TLR2 *	+	+	+	-	12874299
IL7	+	-	+	+	17496983
IL18	+	-	-	+	19467215
EIF2AK2	+	+	+	-	19596385
CD40LG	+	+	+	+	11403919
CD40	+	+	-	-	11403919
CD28	+	+	-	-	12594842
C3	+	+	+	-	19477524
TLR4 **	+	-	-	-	12874299
TP53 **	-	+	-	-	10379742
FCGR2B **	-	+	-	-	12874345
CD46	-	+	+	-	11757799
NCAM1 *	-	-	+	+	16316416
CXCL10 *	-	-	+	-	10799249
CD86 *	-	-	+	-	12594842
HSPD1	-	-	+	-	12218165
IFNA1	-	-	-	+	19667099
CCL2 *	-	-	-	+	19833737
TPBG	-	-	-	+	16630022
GNLY	-	-	-	+	10644038
CD8A	-	-	-	+	18425263

These genes were ranked among the top 20 by at least one of the centrality measures in the literature-mined IFN-γ and vaccine network using VO (i.e. IFNG-vaccine-VO network). The sign “+” means that a gene is ranked among top 20 with the corresponding centrality measure. The sign “-” indicates the opposite. Genes marked with * were not ranked high in the IFNG-vaccine network built without using the VO (i.e. IFNG-vaccine network). Genes marked with ** were not found in the IFNG-vaccine network. The PubMed PMIDs are listed to affirm that the associations mined are verified by literature.

Based on Table 1, three different levels of prediction are available based on the comparison between the IFNG-vaccine network and the more specific IFNG-vaccine-VO network:

(i) Genes ranked high in both networks: 23 genes were ranked high in both the IFNG-vaccine and IFNG-vaccine-VO networks. A more detailed analysis indicated that many of these genes had different levels of centralities (data not shown). It suggests that the roles of certain genes (*e.g.*, IL6) in vaccine research have widely been recognized but studied in more depth in certain vaccines.

(ii) Genes ranked high in the IFNG-vaccine-VO network but not in the IFNG-vaccine network: Six genes (marked with *) are included in this group, *i.e.*, NFKB1, TLR2, NCAM1, CXCL10, CD86, and CCL2. These genes are found in the IFNG-vaccine network, but are not inferred as genes important for vaccine development, although there exists supporting literature evidence (Table 1). Using the VO enabled the identification of these vaccine-related genes.

(iii) Genes ranked high in the IFNG-vaccine-VO network but not found in the IFNG-vaccine network: This group includes three genes (marked with **), *i.e.*, TLR4, TP53, and FCGR2B. These genes are not contained in the IFNG-vaccine network. Using the VO enabled the discovery of these genes as belonging to the IFNG-vaccine mediated gene interaction network and as genes important for vaccine research.

These gene lists provide new information to study vaccine-induced human gene networks associated with IFNG. For example, Toll-like receptor-4 (TLR4) is an important cell receptor that participates in many immune responses against pathogen infections. TLR4-active agents are often developed as vaccine adjuvants [12]. The finding of the presence of TLR4 in the IFNG-vaccine-VO network, but absence from the IFNG-vaccine network is a demonstration that our ontology-based method provides reasonable and useful information to better understand the vaccine-associated immune networks.

### The predicted IFNG-BCG network

**Figure 3:**
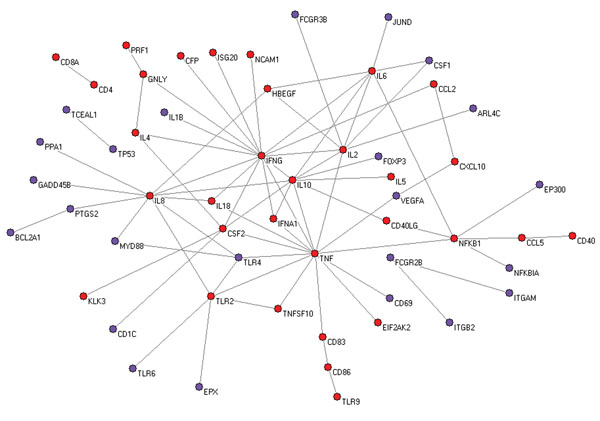
The IFNG-BCG network. All edges represent gene-gene interactions that are associated with the BCG vaccine. In total 24 new genes (colored with purple) are found by using the term “BCG” contained in the VO.

As an example of specific study on a single vaccine, BCG is a licensed tuberculosis vaccine to protect against infection of *Mycobacterium tuberculosis*. We used the “BCG” term and all its synonyms in VO to extract the network of interactions related to the BCG vaccine. The resulting network consists of 56 genes and 77 interactions (Figure 3). In total, 24 of these genes (colored with purple in Figure 3) were not found in the IFNG-vaccine network, which was constructed without using the “BCG” term in the VO.

BCG actively interacts with IFNG and genes in the IFNG network. For example, the interactions between BCG treatment, TLR2 and TLR4 are interesting. BCG is able to activate TLR2 and TLR4 [13]. It induces the maturation of dendritic cells (DCs) via both TLR2 and TLR4 [14]. It can also induce TNF-alpha secretion from DC via TLR2 and TLR4 [15]. Our program also identified TNFSF10 (synonym: TRAIL) and TLR2 that are associated with BCG treatment (Figure 3). It was reported that BCG can directly stimulate the release of tumor necrosis factor (TNF)-related apoptosis-inducing ligand (TRAIL, a synonym for TNFSF10) from polymorphonuclear leukocytes (PMN) through toll-like receptor-2 (TLR2) recognition that is augmented by IFNG [16]. BCG treatment also triggers the induction of FCGR2B (synonym: CD32) on PMN [17], urinary IFNG, IP-10, TNF-alpha, and vascular endothelial growth factor (VEGF) [18]. All these genes and interactions were detected by our program and exist in the IFNG-BCG network.

Many new hypotheses can be generated by comparing the three layers of IFNG networks. For example, a direct interaction between IFNG and TLR6 was not reported as shown in the IFNG-BCG network (Figure 3). However, such interaction has been reported in the literature [19] and shown in the generic IFNG network. TLR6 (a co-receptor for TLR2) is a known receptor for bacterial derivatives. Therefore, it can be hypothesized that BCG will also interact with TLR6. Meanwhile, those new genes and interactions induced by BCG treatment may be possibly inferred to other vaccines (*e.g.*, vaccines for intracellular pathogens such as Influenza vaccines or *Brucella* vaccines). Those genes and networks in the generic IFNG or IFNG-vaccine network may provide new genes and interactions for inferring future BCG mechanism research.

### The networks of gene-vaccine and vaccine-vaccine associations

An abstract-level co-occurrence method was used to identify the specific vaccines that are associated with IFNG and the top ranked 32 genes listed in Table 1. We assume that a gene and a specific vaccine are associated if they appear together in the same abstract. In summary, 28 vaccines (Table 2) were found to be associated with the most central genes in the IFNG-vaccine-VO network. No other vaccines are identified to be associated with these top ranked 32 genes and IFNG. CD46 (a complement regulatory protein) and TPBG (trophoblast glycoprotein) are the two genes (out of the top 32) that don't co-occur with any of the specific vaccines in VO. These two genes were ranked high in the IFNG-vaccine network, built without using VO. It suggests that these two genes have been well studied in the generic vaccine development context, but not in the context of specific vaccines.

**Table 2:** 28 vaccines identified to be associated with the top 32 genes.

Vaccines	Num. genes	Associated genes
***Inactivated viral vaccine***		
Fluzone	4	IFNA1, CD4, CD8A, IL10
Vaxigrip	4	IFNA1, CD4, CD8A, TNF
Engerix-B	3	IFNA1, CD4, IL2
FSME - IMMUN	3	CD4, IL6, TNF
Fluarix	1	IFNA1
Havrix	1	CD4
Vaqta	1	CD4
***Live attenuated bacterial vaccine***		
BCG Vaccine	31	HSPD1, IL18, CCL2, IL5, CD40, EIF2AK2, TLR4, IL13, CD40LG, CD86, IFNG, CSF2, CXCL10, TP53, FCGR2B, NFKB1, IFNA1, GNLY, IL2, IL10, IL4, CD28, CD4, C3, IL6, CD8A, IL8, TLR2, IL7, TNF, NCAM1
LVS	17	CCL2, CD40, TLR4, CD86, IFNG, IFNA1, NFKB1, IL10, IL2, IL4, CD4, C3, IL6, IL8, CD8A, TLR2, TNF
*Brucella* strain 19	5	IFNA1, CD4, CD8A, IL2, IL10
RB51	2	CD4, CD8A
CVD 1207	2	IL4, IL5
CVD 1208	1	IFNG
CVD 1208S	1	IFNG
***Live attenuated viral vaccine***		
Rotarix	2	CD4, CD8A
RotaTeq	2	CD4, CD8A
Varilrix	2	CD4, CD8A
Varivax	1	CD4
ProQuad	1	CD4
***Other live vaccine***		
Dryvax	5	IFNA1, CD4, CD8A, IFNG, IL2
TICE BCG	3	IFNA1, CD4, CD8A
***Subunit vaccine***		
RTS,S/AS02A	7	IFNA1, IL4, CD4, IFNG, IL5, CD8A, IL2
Pneumo 23	3	IFNA1, IL4, CD4
FMP1/AS02A	2	IFNA1, IL5
Gardasil	2	CD4, IL2
ActHIB	1	CD4
Infanrix	1	IFNA1
Menactra	1	C3

**Figure 4:**
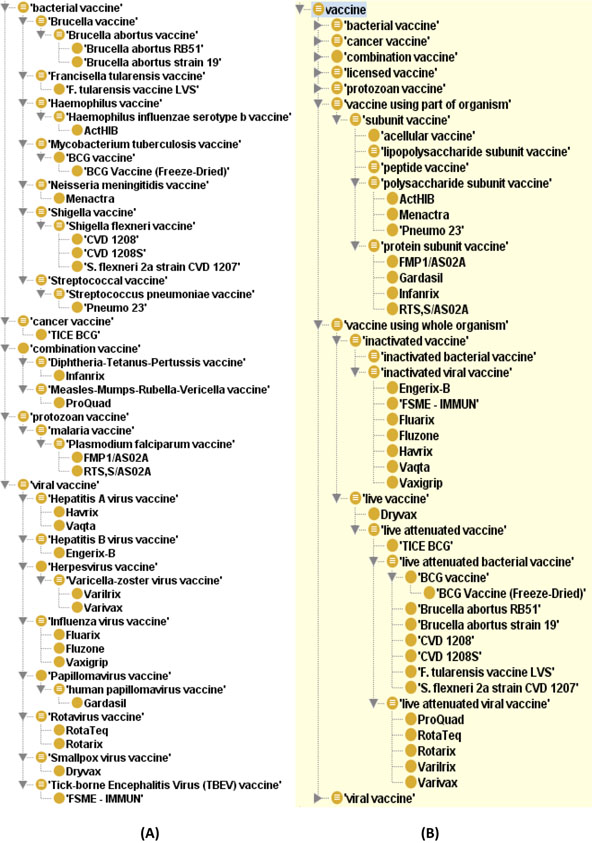
**The 28 vaccines that are associated with the top 32 genes. (A) Asserted hierarchy; (B) Inferred hierarchy after using HermiT OWL reasoner (http://hermit-reasoner.com/). The results were extracted from the Vaccine Ontology using OntoFox**[25]**and displayed using the Protégé ontology editor (http://protege.stanford.edu/).**

Based on the associations between different genes and vaccines and the knowledge of these specific vaccines gained from the VO, it is possible to analyze the associations between different vaccines. For example, different vaccine types can be classified based on the asserted VO vaccine hierarchy (Figure 4A) and inferred VO vaccine hierarchy (Figure 4B). The asserted ontology hierarchy is an ontology hierarchy specified by an ontology editor(s). As an application of the Web Ontology Language (OWL) [20], the inferred ontology hierarchy is generated by a specific ontology reasoner, such as FACT++ (http://owl.man.ac.uk/factplusplus/), based on necessary and sufficient conditions. The combination of necessary and sufficient conditions makes an ontology class a fully defined class. For example, the class term *inactivated vaccine* is fully defined as:

*‘vaccine using whole organism’ and* (*has_quality some inactivated*)

Many vaccines (*e.g.*, Fluarix) have the characteristics (necessary condition) of being inactivated. For example, Fluarix has the following necessary condition:

has_quality some inactivated

Many vaccines also use whole microbial organism for vaccine development. Based on the logic definition of the VO term *inactivated vaccine*, those vaccines (*e.g.*, Fluarix) that are annotated in VO as using whole microbial organism and having the characteristics of being inactivated can be inferred as inactivated vaccines although they are not originally asserted as such (Figure 4).

The vaccines are organized based on the inferred ontology hierarchy in Table 2. More phenomena can be observed by comparing the gene-vaccine network and the vaccine ontology hierarchies. For example, it is shown that the live attenuated bacterial vaccine BCG has been found to interact with 31 out of the top 32 genes described above. Another live attenuated bacterial vaccine also interacts with 17 genes. It is interesting that CD4 is associated with every viral vaccine in the vaccine list (Table 2). Among the 28 vaccines, most subunit vaccines and all viral vaccines, including three Influenza vaccines (Fluarix, Fluzone, and Vaxigrip), are not directly associated with IFNG; however, they are frequently associated with IFNA1 (IFN-alpha 1).

**Figure 5:**
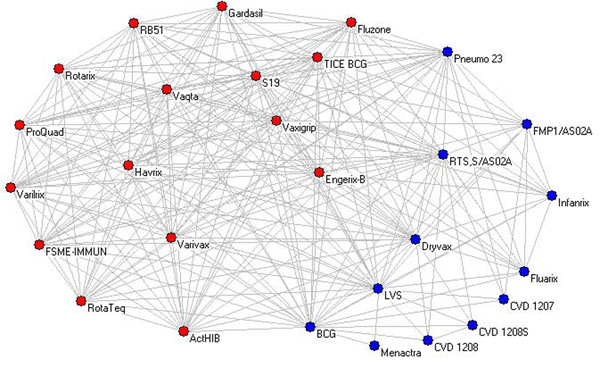
The vaccine-vaccine association network. Vaccines in one of the communities are shown in red and the vaccines in the other community are shown in blue.

A vaccine-vaccine association network was further built, where two vaccines are connected if they share at least one gene. For example, an edge between the vaccines Fluzone and Fluarix is created, since they are both associated with the IFNA1 gene (Table 2). In total, 259 edges were found in the final vaccine-vaccine association network (Figure 5).

A centrality analysis was further performed based on this network. The BCG and LVS have the highest centrality scores by all four centrality methods. These two vaccines are connected with all the other vaccines in the network with at least one shared central gene (Figure 5). The vaccines next to them include RTS,S/AS02A, Dryvax, and Pneumo 23. The vaccines S19, Fluzone, Vaxigrip, TICE BCG, and Engerix-B follow them and share the same centrality scores with each other. These ten vaccines are most widely studied in the context of IFNG research. All the rest are ranked lower with the same score.

The community detection algorithm described in the Methods section identified two vaccine communities that are shown with red and blue in Figure 5. It is interesting to classify the vaccines in these two communities based on the vaccine classification from the Vaccine Ontology (Figure 4). One of the communities (shown in red) consists of mostly viral vaccines. Out of 16 vaccines, this community contains 12 viral vaccines, three bacterial vaccines, and one cancer vaccine. The cancer vaccine (TICE BCG) contains live attenuated BCG bacteria. In contrast, the other community (shown in blue) contains mostly bacterial vaccines. Specifically, this community contains eight bacterial vaccines, two protozoan vaccines, and two viral vaccines. It appears that the top five vaccines with the highest centrality scores (BCG, LVS, RTS,S/AS02A, Dryvax, and Pneumo 23) are located in one single community.

## Discussion

Our study indicates that the application of the Vaccine Ontology (VO) in the centrality-based literature mining enhanced the gene interaction discovery, leading to our finding of new genes and interactions that could not be found before. Our method also allows the generation of the networks of gene-vaccine and vaccine-vaccine associations, leading to better understanding of vaccine-induced immune mechanisms.

The VO is developed using the Web Ontology Language (OWL) [20]. The OWL-based vaccine ontology provides rich semantic constructs, such as necessary and sufficient conditions. An asserted ontology term hierarchy provides user-defined is_a definitions. Each ontology term also includes different ontological attributes, which can be used for ontological reasoning and generating inferred ontology hierarchy (Figure 4B). These hierarchies can be used for ontological analyses of various networks generated using centrality and ontology-based literature discovery

Biomedical ontologies, particularly, the Gene Ontology (GO), have been used in retrieval of gene interaction networks based on literature data [21,22]. For example, Daraselia et al. [21] developed a method for automatic extraction of gene ontology annotation and its correlation with clusters in protein networks. The approach developed by Raychaudhuri et al. [22] assigned GO terms to PubMed abstracts and then assigned identified GO terms to proteins based on statistical analysis of their occurrences in PubMed abstracts. Compared to existing methods, the primary novelty of our ontology-based network discovery approach is that it is based on the novel literature-mined network centrality analysis method that we internally developed [3]. The centrality- and ontology-based approach is generic and can be used to identify other gene interaction networks using biomedical ontologies in other domains.

**Figure 6:**
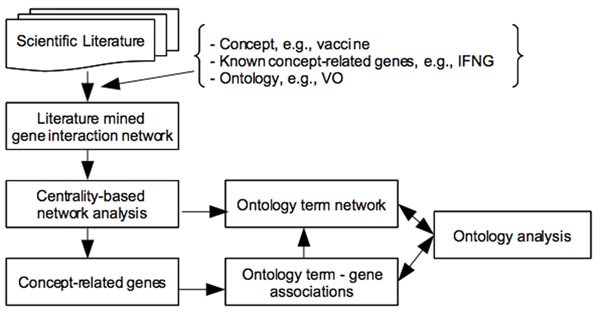
The general framework of the CONDL strategy (a centrality and ontology-based network discovery using literature data).

Derived from current research, we propose a new strategy of a Centrality and Ontology-based Network Discovery using Literature data (CONDL). The general framework of this CONDL strategy is illustrated in Figure 6. Given a concept of interest (*e.g.*, vaccine), an ontology in this concept (*e.g.*, VO), and a set of known concept-related genes (seed genes, *e.g.*, IFNG), the goal is to predict novel concept-related genes. First, a gene interaction network is built by automatically extracting the interactions of the seed genes and their neighbors from the literature. Then, network centrality metrics are used to rank the genes in the network. The underlying hypothesis is that the central genes in this concept-specific network of interactions are also likely to be related to the concept. By comparing the resulting generic gene network (*e.g.*, the generic IFNG network) and the concept-specific subnetwork (*e.g.*, IFNG-vaccine network), many new observations and hypotheses were generated [3]. The ontology can be used to extend the concept-specific subnetwork. Furthermore, a network of gene-ontology term associations, and a network of ontology terms can be generated. The ontological attributes of ontology terms can be used for more advanced ontology analyses (Figure 6).

To further facilitate the study of interaction networks, we have started to generate an Interaction Ontology Network (INO; http://sourceforge.net/projects/ino). INO was initiated to classify those more than 800 interaction keywords we manually collected in this study. These keywords are organized in INO using a hierarchical structure and aligned with the Basic Formal Ontology (BFO; http://www.ifomis.org/bfo). For example, INO includes an ontology class term *increase*, which has synonyms increases, increased, increasing, and elevated. The parent ontology term of *increase* in INO is *positive regulation*. This strategy of classifying interaction terms in hierarchical ontology structure will allow the literature mining and ontology communities to further re-use these terms and support automated reasoning.

Our future plan includes a larger scale of vaccine interaction network analysis beyond the IFNG-associated networks. We are also developing a web server to store the analyzed data and provide a user-friendly web interface to query and visualize the results. The interactions shown in our networks may be specific for certain conditions. The interactions may not be true when experimental conditions change. The possibilities of using other ontologies in our analyses would facilitate our gene interaction network investigations. For example, the Cell Ontology [23] can be used to extract cellular locations of gene interactions. The Cell Line Ontology [24] can be used to determine which cell lines were studied for specific gene interactions.

## List of abbreviations used

GO: Gene Ontology; HGNC: HUGO Gene Nomenclature Committee; HUGO: Human Genome Organisation; LBD: Literature-Based Discovery; NCIBI: National Center for Integrative Biomedical Informatics; OWL: Web Ontology Language; PMN: polymorphonuclear leukocytes; SVM: Support Vector Machine; TLR4: Toll-like receptor-4; TNF: Tumor necrosis factor; TRAIL: (TNF)-related apoptosis-inducing ligand; VO: Vaccine Ontology.

## Competing interests

The authors declare that they have no competing interests.

## Authors' contributions

AO: Project design, software programming, data analysis, and drafting of manuscript; ZX: VO data generation, discussion, and manuscript editing. DRR: Project design and discussion; YH: Project design, data interpretation, and drafting of manuscript.

## Supplementary Material

Additional file 1 **VO Terms** A file in text format containing the 186 VO terms and their synonyms used in this study. The synonyms of a term are separated with “|#|”. Click here for file
